# Laparoscopic Cholecystectomy in a Resource-Constrained Hospital in South Africa: Antibiotic Prophylaxis, Iatrogenic Perforation, Risk Factors, and Management

**DOI:** 10.7759/cureus.76823

**Published:** 2025-01-02

**Authors:** Arvin Khamajeet, Ahmed Diab, Bader Al Taweel, Dilya Luchoo, Fazlin Noor, Heather Bougard

**Affiliations:** 1 Department of General Surgery, New Somerset Hospital/University of Cape Town, Cape Town, ZAF; 2 Department of Hepatobiliary Surgery and Transplant, Centre Hospitallier Universitaire Montpellier/University of Montpellier, Montpellier, FRA; 3 Department of Mathematics and Statistics, University of Mauritius, Pamplemousses, MUS

**Keywords:** antibiotic protocol at cholecystectomy, antibiotics prophylaxis, developing country, gallbladder perforation management, iatrogenic perforation, infectious complications, laparoscopic cholecystectomy, risk factors for gallbladder perforation, suction and lavage, surgical complications

## Abstract

Introduction

Iatrogenic gallbladder perforation is a notable complication of laparoscopic cholecystectomy. Despite its prevalence, optimal antibiotic prophylaxis strategies remain controversial. This study examines the use of antibiotics during laparoscopic cholecystectomy, evaluates the management of gallbladder perforations, and identifies associated risk factors.

Method

A retrospective analysis of 152 laparoscopic cholecystectomy patients at New Somerset Hospital was conducted, covering April 2021 to June 2023. Data collection included demographics, comorbidities, surgical indications, imaging findings, antibiotic regimens, and intraoperative and postoperative management of gallbladder perforations. Histological outcomes and complication management were also reviewed.

Results

Among the 152 patients, 86.85% (n=132) were female. Gallbladder perforation occurred in 55.9% (n=85). Spillage findings varied: bile alone (n=59), bile with stones (n=19), stones alone (n=3), pus (n=1), and stone with pus (n=1). Two patients had no spillage after iatrogenic perforation. Intraoperative management universally involved suction and lavage. Sixteen antibiotic protocols were documented, with 140 patients receiving preoperative, intraoperative, postoperative antibiotics, or combinations thereof. No infectious complications were reported. Age over 40 (65.2%, n=92) was a significant risk factor for perforation (p<0.05).

Conclusion

This study recommends a single preoperative dose of cefazolin to prevent infectious complications, even in cases of gallbladder perforation. Suction, washout, and retrieval of spilled stones are essential for managing perforations effectively. Establishing standardized antibiotic protocols could improve outcomes and reduce variability in clinical practice.

## Introduction

Laparoscopic cholecystectomy has emerged as the preferred approach for gallbladder surgery due to its favorable outcomes in terms of mortality, morbidity, and hospital stay [1,2]. However, iatrogenic gallbladder perforation remains a notable complication, with an incidence reported at approximately 16% to 31% depending on studies [3-6]. The Scottish national practice guidelines do not recommend routine antibiotic prophylaxis for laparoscopic cholecystectomy [7]. A meta-analysis by Pasquali et al. in 2015, showed that antibiotic prophylaxis was not required for low-risk cholecystectomies [8]. In support of these guidelines and studies is a recent meta-analysis by Evans et al. in 2021, which showed that iatrogenic gallbladder perforation was not linked to collections or surgical site infections [9]. However, there have been some controversies regarding this practice due to results obtained in other systematic reviews and meta-analyses. Indeed, Kim et al. demonstrated in their meta-analysis in 2018 that prophylactic antibiotics can help prevent deep-seated surgical site infections [10]. Similarly, Matsui et al. in their re-appraisal of the previously reported meta-analysis, in 2017, reported that antibiotic prophylaxis reduces surgical site infection as well as distant infections [11].

The controversy surrounding iatrogenic gallbladder perforation and the spillage of stones or bile lies in its optimal management. A recent large cohort study conducted in Sweden found that antibiotic administration following iatrogenic perforation did not significantly impact outcomes, but the perforation itself was associated with increased complications [12]. Conversely, Karabulut et al. suggested that a single dose of antibiotics suffices to prevent infectious complications related to iatrogenic perforation [13]. Moreover, Hui et al. in their study in 1999 suggested that the risk of late abscess formation was not increased in patients with iatrogenic gallbladder perforation, even after a follow-up period of approximately four years [5].

The rise of antibiotic resistance presents a significant challenge in modern medicine, and appropriate antibiotic usage is crucial in combating this issue. Studies conducted in Bangladesh and elsewhere have demonstrated that irrational antibiotic use contributes to the escalation of resistance rates [14,15].

To date, there is a lack of data within our institution, New Somerset Hospital, and other facilities in Cape Town regarding iatrogenic gallbladder perforation and its intraoperative and postoperative management, particularly regarding antibiotic prophylaxis.

The aim of our study was to assess the rate of iatrogenic perforation and the utilization of antibiotics, to determine whether antibiotic administration impacts postoperative outcomes, to assess any factors that might be impacting the rate of perforation, and to investigate our management of iatrogenic perforation.

## Materials and methods

Design and participants

A retrospective review of all patients who had a laparoscopic cholecystectomy at New Somerset Hospital, situated in the Western Cape province of South Africa, was performed for patients who received the surgery between April 2021 and June 2023. The study received approval from the Human Research Ethics Committee with approval number 545/2023. Patients who received surgery during the study period were identified using our operation schedule database. Data was collected from hospital folders, National Health Laboratory Service (NHLS) histopathology reports and the hospital Picture Archiving and Communication System (PACS). Patient demographics collected were age and gender at presentation. Reasons for cholecystectomy were also recorded. Perforation data, if any, was obtained from the surgeon's theatre notes, and perforation content, whether it be pus, bile, or stone that was spilled, was recorded. No culture is taken in our setting. Information about pre-op and intra-op antibiotics used was obtained from the anesthetic records while any use of post-op antibiotics, if prescribed, was obtained from prescription charts. Any complications and management thereof were recorded as well.

Statistics

Descriptive statistics were employed to provide an overview of patient demographics and underlying pathology. Parametric data, which adhered to the normal distribution, were presented as means accompanied by standard deviation and range values. Conversely, non-parametric data, which did not meet the assumptions of normality, were described using the median and interquartile range to capture the central tendency and dispersion of the data accurately. Pearson Chi-square test was used to calculate the p-value. A p-value <0.05 was considered statistically significant.

## Results

A total of 164 patients underwent cholecystectomy between April 2021 and June 2023. Twelve patients were excluded due to incomplete data, resulting in a final cohort of 152 patients. The demographic and medical characteristics of the study population are presented in Table 1.

**Table 1:** Demographics of patients with Risk factors and their correlation with iatrogenic perforation of gallbladders at laparoscopic cholecystectomy

Risk factor	Overall	Perforation	No perforation	P-value (Pearson test)	Chi-square statistic
Age	N=152	N=85	N=67	0.004	8.17
<40	60 (39.47%)	25(29.4%)	35(52.2%)		
>40	92 (60.53%)	60(70.6%)	32(47.8%)		
Gender					
Gender	N=152	N=85	N=67	0.69	0.16
Female	132 (86.85%)	73(85.9%)	59(88.1%)		
Male	20 (13.15%)	12(14.1%)	8(11.9%)		
No of comorbidities					
No of comorbidities	N=152	N=86	N=66	0.72	0.65
0	77(50.65%)	46	31		
1	37 (24.35%)	20	17		
>1	38 (25%)	20	18		
BMI					
BMI (kg/m2)	N=132	N=72	N=60	0.13	2.25
>40	25 (18.93%)	17(23.6%)	8(13.3%)		
<40	107 (81.07%)	55(76.4%)	52(86.7%)		
HIV					
HIV	N=152	N=85	N=67	0.30	1.07
Positive	16 (10.52%)	7(8.2%)	9(13.4%)		
Negative	136 (89.48%)	78(91.8%)	58(86.6%)		
Diabetes Mellitus (DM)					
DM	N=152	N=85	N=67	0.51	0.44
Yes	14 (9.21%)	9(10.6%)	5(7.5%)		
No	138 (90.79%)	76(89.4%)	62(92.5%)		
Hypercholesterolemia					
Hypercholesterolemia	N=152	N=75	N=67	0.80	0.065
Yes	17 (11.18%)	10(13.3%)	7(10.4%)		
No	135 (88.82%)	75(86.7%)	60(89.6%)		
96 (63.2%) samples were not sent for histology					
Histology	N=56	N=32	N=24	0.57	3.83
Chronic cholecystitis	41 (73.2%)	23(71.8%)	18(75%)		
Acute on chronic cholecystitis	7 (12.5%)	5(15.6%)	2(8.3%)		
Xanthulomatous cholecystitis	4 (7.1%)	3(9.4%)	1(4.2%)		
Subacute cholecystitis	2 (3.6%)	1(3.1%)	1(4.2%)		
Chronic cholecystitis + intestinal metaplasia	1 (1.8%)	0	1(4.2%)		
Xanthulomatous cholecystitis + acute on chronic cholecystitis	1 (1.8%)	0	1(4.2%)		
Ultrasound findings					
Ultrasound findings	N=148	N=83	N=65	0.21	3.08
Previous choledocholithiasis	28 (18.9%)	18(21.7%)	10(15.4%)	0.33	
Cholelithiasis	86 (58.1%%)	43(51.8%)	43(66.2%)	0.08	
Previous cholecystitis	34 (23.0%)	22(26.5%)	12(18.5%)	0.25	

Of our study population, 132 (86.85%) were female, with a median age of 33.9 years. Notably, perforation was detected in 85 patients (55.9%).

Risk factors for gallbladder perforation

Age emerged as a significant risk factor for gallbladder perforation in our study. 60 individuals (39.47%) were below 40 years old with 29.4% (n=25) of those patients having a gallbladder perforation, while 92 (60.53%) were over 40 and 70.4% (n=60) of these patients had a gallbladder perforation. Of note, the age of 40 was chosen due to anecdotal evidence from local surgeons that above 40 years old, they thought there was a lot of iatrogenic perforations. Our results demonstrated statistical significance for age greater than 40 years (p-value=0.004).

Furthermore, BMI was not identified as a significant risk factor. Among 132 patients with recorded BMI, 81.07%(n=107) had a BMI <40, with 76.4%(n=55) of them experiencing perforations. In contrast, 18.93% (n=25) patients had a BMI >40, among whom 23.6%(n=17) had gallbladder perforations (p-value=0.13). The BMI was arbitrarily chosen on the basis of grade 3 obesity being a BMI greater than 40.

Gender difference did not emerge as a statistically significant risk factor (p-value=0.69).

The analysis of comorbidities revealed no statistically significant association with gallbladder perforation (p-value=0.72). Among the patients, 50.65% (n=77) had no comorbidities,24.35% (n=37) had one comorbidity, and 25%(n=38) had more than one comorbidity. 46 (59.74%) patients with no comorbidity had a perforation, 20 (54.05%) patients with one comorbidity had a perforation, and 20 (52.6%) patients with more than one comorbidity had a perforation.

Regarding specific comorbidities, 16 patients (10.52%) were diagnosed with HIV, among whom seven experienced gallbladder perforation. Similarly, 14 patients (9.21%) had diabetes mellitus (DM), and nine of them encountered gallbladder perforation. Additionally, 17 patients (11.18%) had a history of hypercholesterolemia, with 10 of them experiencing gallbladder perforation. However, none of these comorbidities demonstrated a statistically significant association with gallbladder perforation.

Intraoperative findings

The patients with perforated gallbladders had the following spillage findings: 69,4% (n=59) patients had bile, 22.4% (n=19) had bile and stones, 3.5% (n=3) patients had stones, 1.2%(n=1) patient had pus, 1.2%(n=1) patient had stone and pus, and finally, two had nothing.

Intraoperative management of perforated gallbladder

Of the 85 patients with perforation, we only had data for 69 patients regarding their intraoperative management. 67 (n= 67,97.1%) patients had a normal saline wash and suction, 1.4% (n=1) had suction only, and 1.4% (n=1)had suction with an abdominal drain left in situ as well.

Postoperative histology

Our study analysis revealed that a significant portion of patients, 63.2% (n=76), did not undergo histopathological examination, thereby indicating a common practice of omitting this step in our hospital. Among the patients who did undergo histological analysis, comprising 36.8% (n=56) cases, the most prevalent finding was chronic cholecystitis, accounting for 73.2% (n=41) of cases. This was followed by acute on chronic cholecystitis at 12.5% (n=7) and xanthogranulomatous cholecystitis at 7.1% (n=2).

However, none of the histopathological observations demonstrated statistical significance as a risk factor for gallbladder perforation (p-value=0.57). Interestingly, the distribution of these histological findings followed a similar pattern in both the perforated and non-perforated patient groups, indicating a lack of correlation between histopathological characteristics and the occurrence of gallbladder perforation.

Ultrasound findings

Among the patients, 97.4% (n=148) had ultrasound findings, namely 58.1%(n=81) demonstrating cholelithiasis as the most common finding, 23.0%(n=34) cholecystitis, and lastly, 18.9% (n=28) had choledocholithiasis. However, none of the ultrasound findings were statistically significant in relation to gallbladder perforation.

Antibiotic regimen

The spectrum of prescribed antibiotic regimens exhibited considerable diversity across surgical practitioners, with our investigation identifying a total of 16 distinct protocols. As shown in Table 2, these regimens were meticulously categorized into three delineated phases: preoperative, intraoperative, and postoperative antibiotic administration.

**Table 2:** Antibiotic regimens used during laparoscopic cholecystectomy in our hospital Cefzol is also known as cefazolin; 'Nil' means no antibiotics were used.

Regimen	Pre op Antibiotics	Intra op antibiotics	Post op Antibiotics	Complication
1	Nil	Augmentin	Nil	
	9	9	9	0
Regimen 2				
2	Nil	Augmentin	Augmentin	
	2	2	2	0
Regimen 3				
3	Nil	Flagyl	Nil	
	1	1	1	0
Regimen 4				
4	Nil	Cefzol	Nil	
	9	9	9	1 x Non-infectious subhepatic collection
Regimen 5				
5	Nil	Nil	Nil	
	12	12	12	0
Regimen 6				
6	Nil	Nil	Augmentin	
	1	1	1 x 5 days	0
	1	1	1 x 1 day	0
Regimen 7				
7	Nil	Nil	Ceftriaxone/ampicillin	
	1	1	1 x 3 days	0
Regimen 8				
8	Cefzol	Nil	Nil	
	36	36	36	0
Regimen 9				
9	Cefzol	Nil	Ampicillin/gentamicin	
	1	1	1 x 4 days	0
Regimen 10				
10	Cefzol	Nil	Nil	
	36	36	36	0
Regimen 11				
11	Cefzol	Flagyl	Nil	
	6	6	6	0
Regimen 12				
12	Cefzol	Augmentin	Nil	
	1	1	1	0
Regimen 13				
13	Cefzol	Augmentin	Augmentin	
	1	1	1	0
Regimen 14				
14	Augmentin	Nil	Nil	
	1	1	1	0
Regimen 15				
15	Cefzol/ampicillin	Augmentin	Cefzol/ampicillin	
	1	1	1 x 3 days	1 non-infectious subhepatic collection
Regimen 16				
16	Cefzol/ampicillin	Nil	Cefzol/ampicillin	
	1	1	1	0

Of our study cohort comprising 152 patients, it is noteworthy that 7.89% (n=12) of patients did not receive any antibiotic intervention throughout the entirety of their hospitalization. Among the remaining patients, preoperative antibiotic prophylaxis was administered to 83 individuals (54.60%), with cefazolin emerging as the predominant choice, accounting for 52.6% of prescriptions. Intraoperative antibiotic coverage was provided to 30 patients, predominantly with augmentin, accounting for 46.67% of cases. Furthermore, it is of interest to highlight that cefazolin was also employed intraoperatively in 30% of patients who had not received any preoperative antibiotic prophylaxis, particularly following gallbladder perforation incidents. Postoperative antibiotic therapy was prescribed for a minority of cases, with nine patients receiving such treatment, predominantly comprising augmentin, which accounted for 55.56% of these instances.

Complications

There were two cases of postoperative subhepatic collection, both non-infected. Both cases were successfully treated with percutaneous drainage without the need for antibiotics. Notably, there were no bacteria seen on microscopy or on culture as assessed by the National Health Service (NHS) results.

## Discussion

In our study, we observed a notably high incidence of gallbladder perforation, recorded at 56.4%. This contrasts with findings from previous studies, which reported rates ranging from approximately 16% to 31% [3-6]. A potential contributing factor to this discrepancy may be the prevalence of chronic cholecystitis among our patients, complicating dissection and surgical procedures. It is worth noting that a significant proportion of our patient cohort underwent surgery after prolonged waiting periods, attributable to COVID-19-related delays and limited operating theatre availability.

Our investigation underscores the absence of standardized antibiotic regimens within our surgical unit for laparoscopic cholecystectomy and iatrogenic perforated gallbladders. Among the 16 distinct regimens identified, all were found to be equally effective in preventing postoperative infectious complications.

The decision to forego prophylactic antibiotic administration aligns with findings from two meta-analyses by Pasquali et al. (2016) and Al-Ghnaniem et al. (2003), which concluded that antibiotic prophylaxis may be unnecessary in low-risk patients with a history of biliary colic or previous cholecystitis [8,16]. Similarly, other studies, including a large-scale registry analysis by Jaafar et al. (2021), support the approach of not using preoperative antibiotics in low-risk laparoscopic cholecystectomy patients [17,18]. However, conflicting evidence from studies such as that by Matsui, et al. (2018) and Liang et al. (2015) suggests potential benefits of prophylactic antibiotics in reducing surgical site infections [11,19].

Furthermore, Matsui et al.'s meta-analysis in 2018 re-evaluated previously conducted studies and demonstrated that preoperative antibiotics had a positive impact on distant infection, surgical site infection, as well as overall infections [11]. On the other hand, a meta-analysis by Kim et al in 2018 also found a positive benefit of antibiotics in reducing superficial surgical site infections [10].

The second pertinent finding was that our intraoperative management of gallbladder perforation involved a mixture of wash and suction. This practice is concurrent with the findings of other studies that advocated irrigation of iatrogenic gallbladder perforation with bile spillage [20,21], while Amin et al. showed that the pre- and postoperative course of iatrogenic perforated gallbladders is the same as those with no perforation as long as the aspirate is irrigated and suctioned and an attempt to retrieve all the stones was made [22]. Indeed, Edergren et al. also found an increased risk of infection when the spillage was incompletely cleaned [12]. Of note, this rate ranged between 2.4% and 4.7%, as evidenced by Edergren et al. and Memon et al., and required minimal treatment [12,23].

Additionally, our study identified age greater than 40 as a statistically significant risk factor for gallbladder perforation (p-value <0.05). This finding aligns with prior research by Hama Tofiq et al. and other studies, underscoring the importance of considering the impact of age as a risk factor in assessing surgical risk [3,6]. However, there remains a scarcity of studies examining the statistical significance of age as a risk factor for gallbladder perforation, highlighting the need for further investigation in this area.

The number of comorbidities and the patient’s gender profile had no impact on gallbladder perforation. Likewise, HIV status, dyslipidemia, and diabetes were not found to be statically significant risk factors causing perforation.

It is important to state that gender has been found to be a major risk factor in numerous studies [20,24,25]. In a matched-cohort analytic study performed by Sarli et al. there was a significantly higher percentage of male patients in the group with an iatrogenic gallbladder perforation [24]. Rice et al. reported that the Iatrogenic gallbladder perforation group had a higher number of male patients (43%) while Mohiuddin et al. in a multivariate logistic regression analysis found that male sex is an independent risk factor for gallbladder perforation (p<0.05) [20,25].

Diabetes was not a clear risk factor for perforation in our study. In a study by Hama Tofiq et al., the most frequent chronic disease among patients who had iatrogenic gallbladder perforation was diabetes mellitus (DM) in 18.2% [6]. Urakawa et al. also found an increased risk of complications in diabetic patients [26]. There seems to be an indirect relationship with gallbladder perforation, as most diabetic patients have a high BMI. Berbudi et al. suggested that these patients often have silent gallbladder disease and present in situations such as acute inflammation, empyema, or gangrenous gallbladder [27].

There is not much data in the literature about HIV, hyperlipidemia, and the number of comorbidities as risk factors for iatrogenic perforation.

A notable observation in our study was that a majority of gallbladder specimens, accounting for 63.2% of all the specimens, were not sent for histopathological examination. Upon histological analysis of the specimens that were examined, the primary findings included chronic cholecystitis, acute on chronic cholecystitis, and xanthogranulomatous cholecystitis, and these histopathological results had no impact on the rate of gallbladder perforation in our study. Due to the high percentage of gallbladders not sent for analysis, proper statistical analysis is very difficult. Our practice is guided by studies such as Bastiaenen et al. and Koppatz, et al., which found very low rates of gallbladder cancer of 0.5% and 0.04-0.08%, respectively, where surgeons found the gallbladder to be macroscopically normal on clinical examination after surgery [28,29]. Our practice is guided by cost-cutting measures as a result of resource constraints.

The limitation of this study is the small size of the sample, which may impact some of the statistical significance of the findings. It is also to be noted that the surgeon as a risk factor has been omitted, mainly as a result of the sample size, which might have introduced bias.

## Conclusions

In conclusion, in a developing country like South Africa, which has limited resources, a single dose of cefazolin should be enough as a preoperative antibiotic in all laparoscopic cholecystectomies, in view of the results of more recently published meta-analyses. One should also be more careful while performing dissection in older male patients, and whenever there is any perforation, attempts should be made to aspirate and wash any spillage and retrieve any stones. In so doing, the risk of complications as well as the cost of managing the patient will be reduced.
